# 556. Commercial Microbial Cell-Free DNA Next-Generation Sequencing in a Pediatric Quaternary Care Facility Yields Limited Diagnostic Utility

**DOI:** 10.1093/ofid/ofad500.625

**Published:** 2023-11-27

**Authors:** Lucas J Osborn, Dina Kamel, Sanchi Malhotra, Leila C Posch, Cristina Costales, Jennifer Dien Bard

**Affiliations:** Children's Hospital Los Angeles, Los Angeles, California; Children's Hospital Los Angeles, Los Angeles, California; Children's Hospital Los Angeles, Los Angeles, California; Children's Hospital Los Angeles, Los Angeles, California; Children's Hospital Los Angeles, Los Angeles, California; Children’s Hospital Los Angeles, Los Angeles, California

## Abstract

**Background:**

Microbial cell-free DNA next-generation sequencing (mcfNGS) is a novel diagnostic tool increasingly utilized as a rapid, noninvasive approach to aid in the diagnosis of infectious diseases. However, its utility in pediatric patients is not well established. This study aimed to explore the diagnostic utility of commercial microbial cell-free DNA sequencing in a pediatric quaternary care facility.

**Methods:**

This retrospective study included 15 patients with mcfNGS results between June 2018 and April 2023. Chart abstraction was performed to determine changes in clinical management. Change in management was defined as any clinical decision implemented based on mcfNGS results. Overall clinical impact was defined as either a beneficial or deleterious change in patient outcome resulting from clinical management changes made based on mcfNGS results.

**Results:**

Of the 15 patients, a total of 14 valid results were reported, 13 (86.7%) of which were from the first attempt. At least one microbial target was detected in 10/14 (71.4%) patients. Standard microbiology testing (SMT) revealed a pathogen or diagnostic marker of such in 8/14 (57.1%) patients. Notably, there were no instances in which mcfNGS detected a true pathogen in the context of negative SMT. Of the 14 patients with valid reports, clinical management was changed based on mcfNGS in 3 (21.4%) patients. This included addition of anti-fungal therapy, de-escalation of antimicrobials, and initiation of immunosuppressive corticosteroid therapy in the context of a negative mcfNGS result. Of the 10 patients in which at least one microorganism was detected, 70% were determined to be either contamination or of unclear clinical significance. There was only one instance in which mcfNGS alone led to a beneficial clinical impact as determined by de-escalation of anti-fungal therapy.

Figure 1.

**Figure:**
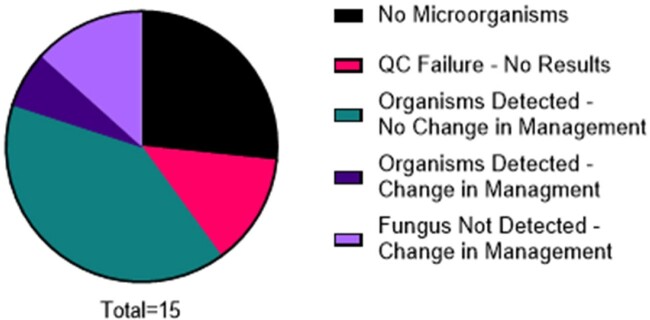

mcfNGS was performed on 15 pediatric patients between July 2018 and April 2023. Pie chart represents categorization of mcfNGS results and their clinical impact.

**Conclusion:**

While infectious disease metagenomic NGS holds great promise, the utility of currently available commercial assays for non-invasive specimens remains poorly understood. Here we report that in a small cohort of pediatric patients, commercial mcfNGS added minimal value over standard microbiological testing. Additional investigation is required to determine the ideal clinical picture warranting mcfNGS.

**Disclosures:**

**Jennifer Dien Bard, PhD**, Abbott Molecular: Grant/Research Support|BioMerieux: Advisor/Consultant|BioMerieux: Grant/Research Support|BioMerieux: Honoraria|Genetic Signature: Advisor/Consultant|Genetic Signature: Grant/Research Support|Luminex: Grant/Research Support|Salve: Stocks/Bonds|Thermo Fisher: Honoraria

